# The Genetic Integrity of the *Ex Situ* Population of the European Wildcat (*Felis silvestris silvestris*) Is Seriously Threatened by Introgression from Domestic Cats (*Felis silvestris catus*)

**DOI:** 10.1371/journal.pone.0106083

**Published:** 2014-08-27

**Authors:** Kathrin A. Witzenberger, Axel Hochkirch

**Affiliations:** 1 Zoo Hoyerswerda, Hoyerswerda, Germany; 2 Trier University, Department of Biogeography, Trier, Germany; Texas A&M University, United States of America

## Abstract

Studies on the genetic diversity and relatedness of zoo populations are crucial for implementing successful breeding programmes. The European wildcat, *Felis s. silvestris*, is subject to intensive conservation measures, including captive breeding and reintroduction. We here present the first systematic genetic analysis of the captive population of *Felis s. silvestris* in comparison with a natural wild population. We used microsatellites and mtDNA sequencing to assess genetic diversity, structure and integrity of the ex situ population. Our results show that the ex situ population of the European wildcat is highly structured and that it has a higher genetic diversity than the studied wild population. Some genetic clusters matched the breeding lines of certain zoos or groups of zoos that often exchanged individuals. Two mitochondrial haplotype groups were detected in the in situ populations, one of which was closely related to the most common haplotype found in domestic cats, suggesting past introgression in the wild. Although native haplotypes were also found in the captive population, the majority (68%) of captive individuals shared a common mtDNA haplotype with the domestic cat (*Felis s. catus*). Only six captive individuals (7.7%) were assigned as wildcats in the STRUCTURE analysis (at K = 2), two of which had domestic cat mtDNA haplotypes and only two captive individuals were assigned as purebred wildcats by NewHybrids. These results suggest that the high genetic diversity of the captive population has been caused by admixture with domestic cats. Therefore, the captive population cannot be recommended for further breeding and reintroduction.

## Introduction

Due to the ongoing threats to biodiversity, species conservation is still a challenging task [1]. Although in situ conservation represents by far the most effective way to protect endangered species, it is evident that not all species can be preserved in their natural habitats. Therefore, ex situ conservation and reintroductions have become common measures of species conservation [2], [3]. Despite the great advances in organization and logistics (studbooks, online databases) and increasing expertise in veterinary medicine, inbreeding and outbreeding still pose serious threats for ex situ conservation [4]–[7]. Captive breeding may be compromised by unknown founder origin and relationships [8], undetected hybridisation [9], limited representation of natural genetic diversity [10] or studbook errors [11], [12]. These problems can affect both the genetic integrity of the captive population as well as the success of reintroduction projects. Therefore, data on the genetic diversity and relatedness in zoo populations can provide valuable information for improving conservation programmes [13].

The influence of captive breeding on the genetic diversity of endangered species has already been subject to a number of theoretic and genetic studies [7], [14]–[16], [17]. However, the majority of the genetic studies focus on highly endangered species, for which studbooks or international breeding programmes already exist [18]–[20]. This circumstance has two drawbacks: (1) Species with small population size might have a reduced genetic diversity even in their wild populations. Therefore, the genetic diversity of captive populations of such highly endangered species might not reflect the effects of captive breeding. (2) Studbooks are usually kept in a way to avoid the loss of genetic diversity or inbreeding depression. Hence, it is likely that genetic analyses of ex situ populations will confirm the success of such coordinated breeding schemes [16], [21]. However, studbooks exist only for ca. 850 species (WAZA 2010), while ca. 10.000 species are currently kept in zoos and bred without any coordination (ISIS 2010). Many of these species are still rather common in the wild, but nevertheless some of them are regionally endangered and sometimes even subject to reintroductions. In order to assess the influence of uncoordinated breeding on the genetic diversity of a captive population, it is useful to study one of these species rather than well managed captive populations. Comparing the genetic variability of ex situ populations and wild populations also requires that a target species has viable wild populations and a sufficiently large captive population. Furthermore, it should have been successfully bred for many generations in captivity as genetic erosion may accumulate over time [18].

The European wildcat (*Felis silvestris silvestris*) is an ideal study object for such an analysis. It is still rather common in Europe and classified as Least Concern on the IUCN Red List of Threatened Species [22]. However, the populations of this species are fragmented and in some countries declining [22]. Wildcats are kept and bred in many European zoos and captive individuals have already been reintroduced in three regions in Germany [23]. However, to date no coordinated breeding programme or studbook exists for the European wildcat. Hence, there is no information available on the captive stock concerning founder size, founder origin, captive population size and relatedness. As it is planned to establish a studbook for the European wildcat in the near future (A. Sliwa pers. comm.), a genetic analysis of the breeding stock can provide important basic information for this studbook. Another challenge in the conservation of the European wildcat is hybridization with domestic cats, which is a serious threat in some wild populations [24]–[26]. Thus, an additional advantage of a genetic analysis prior to coordinated breeding is the exclusion of potential hybrids.

Here, we present the first systematic genetic analysis of the European captive population of *Felis s. silvestris*. We used both microsatellites and mitochondrial DNA (mtDNA) sequencing in order to assess the genetic variability, structure and integrity of the *ex situ* population. We also analysed samples from a wild population to evaluate if the genetic variability in the captive population is comparable to the diversity found in the wild.

## Methods

### Sampling and DNA extraction

We contacted 124 zoos, 64 of which confirmed keeping European wildcats. We obtained 80 samples (12 buccal swabs, 8 tissue samples from deceased individuals, 10 blood samples and 50 hair samples) from 30 zoos spanning six European countries (see Text S2 in File S1 and Tab. S4 in File S1 for an overview). All ex situ samples were taken during routine veterinary treatments (like vaccination, or the placement of transponders for juveniles) or whenever a veterinary treatment was necessary. The collection of hair samples does not pose a severe stress or hurt to the cats, the same is true for buccal swaps from young individuals. Blood samples were only taken from anaesthetized individuals.

The in situ sample consisted of 89 individuals (26 tissue samples and 63 hair samples) from a natural wild population in the Harz mountains (Germany). The hair samples were collected in the context of a radio telemetry study (M. Götz). The tissue samples were from a monitoring program for roadside casualties. Both studies were carried out with the permit of the Landesamt für Umweltschutz Sachsen-Anhalt which included the collection of samples from European Wildcats (see Text S1 in File S1 for further information). Additionally, we received six samples (3 tissue and 3 hair samples) from road kills and live traps collected in the wild populations in Rhineland-Palatinate and Saarland.

Furthermore, we sampled 33 domestic cats with the permission of their owners, in order to detect hybrids. 15 of the domestic samples came from a private animal shelter which was located near the in situ population. We chose this shelter, as it would provide a realistic insight into the potential influence local feral cats might have on the wild population. For the remaining samples we contacted private owners. The private owners tore out a small bunch of hair, so that the sample included the follicles. This bunch was then sent or given to us in an envelope.

DNA was extracted from buccal swabs, tissue and blood using the DNeasy blood and tissue kit (Qiagen) following the manufacturer's instructions (with special protocols for blood and buccal swabs as provided by the manufacturer). For the hair samples, we extracted DNA with a modified Chelex 100 protocol, using a 10% Chelex solution with addition of 7 µl Proteinase K (18 mg/ml) per sample and overnight lysis [27], [28]. No DNA could be extracted from seven of the 208 samples (5 from wild populations and 2 from the captive population). The respective samples from the wild population were hair samples which had been taken several years ago and had then been stored at room temperature. The captive samples had probably been conserved in denatured ethanol.

### Genotyping

The six samples from the wild caught individuals in Saarland and Rhineland-Palatinate were not genotyped, as the samples size from these populations was too small for population genetic analysis. They served as reference samples for the mtDNA analysis (see below). Therefore only 194 of the 201 samples were genotyped at ten di-nucleotide repeat microsatellite loci (FCA08, FCA23; FCA43; FCA58, FCA77, FCA78, FCA90, FCA126, FCA132 and FCA149) characterized in the domestic cat [29], [30]. Amplification was performed in a Mastercycler (Eppendorf) using the 2.5×5PRIME HotMasterMix (5PRIME). For each PCR we used 5 µl reaction mix containing 1.2 µl genomic DNA, 2.2 µl HotMasterMix, 2.2 µl water and 0.1 µl forward and reverse primers. The PCR conditions were as recommended by the manufacturer, with an annealing temperature of 55°C for most primers (exceptions: FCA90: 60°C; FCA78: 50°C and addition of 2.5 mM Mg^2+^). The 5′-end of each forward primer was labelled with a fluorescent dye, either 5-FAM, TAMRA or JOE. The products were genotyped on a MegaBACE 1000 automated DNA sequencer (GE Healthcare). Fragment lengths were determined using Fragment Profiler 1.2 (Amersham Biosciences). To minimize genotyping errors due to low DNA concentrations (especially in the hair samples), we applied a multiple tube approach as recommended by Taberlet et al. [31]. Samples were only included into further analyses, if they yielded unambiguous results in three independent replications (190 samples met this criterion, Tab. S4 in File S1). Two captive samples had identical genotypes in all three replications and it turned out that this individual had been transferred to another zoo and sampled twice. Therefore, the duplicate sample was excluded from further statistical analysis and this left 189 samples for analysis.

### Sequencing

For 70 wild, 66 captive and 33 domestic individuals we sequenced the mitochondrial DNA fragment NADH dehydrogenase subunit 5 (ND5) based on the study by Driscoll et al. [32]. Most juveniles of sampled mothers and siblings were excluded and later assigned to the same mtDNA haplotype as all sequenced juveniles had the same haplotype as their dam. We finally obtained 88 sequences of wild, 77 of captive and 33 of domestic individuals (Tab. S4 in File S1). The primers CD-ND56-F1C and CD-ND56-R4 [32] were used for amplification in a Multigene Gradient Thermal Cycler (Labnet). We used the 5PRIME HotMasterMix (5PRIME) for amplification. The PCR product was purified using the High pure PCR product purification kit (Roche) according to the manufacturer's protocol. Sequencing was performed with the DYEnamic ET terminator cycle sequencing kit (GE Healthcare) for sequencing reactions run on a MEGABACE 1000 automated sequencer (GE Healthcare). Base-calling was performed in Sequence Analyzer 4.0 (Amersham Biosciences).

### Data analysis

#### Genotyping

As the integration of hybrids and genetic sub-groupings were unknown, we first searched for genetic structure in our samples using Structure 2.3.4 [33]. We assumed admixture between groups and used the correlated allele frequency model with a burn-in period of 100,000 simulations, followed by one million Markov chain Monte Carlo simulations. Tests were run for K = 1-15 with ten iterations for each K. In order to detect potential hybrids between wildcats and domestic cats, we used the STRUCTURE results at K = 2 and assigned all individuals either to the cluster “wildcats”, “domestic cats” or “hybrid” [34]. The “hybrid” cluster was chosen, if Q values varied between 0.2 and 0.8 as recommended by Randi and Schulte et al. [35], [36]. The optimal values for K were assessed using both the method described by Pritchard et al. [33], and the method suggested by Evanno et al. [37]. The method described by Evanno et al. [37] tends to result in low K values [38], [39] and generally works better for scenarios with strong genetic differentiation [40] as it detects the highest level of differentiation. The method described by Pritchard et al. [33] might lead to inflated K-values if closely related individuals exist in the sample. Therefore, the clusters might more likely represent family groups and lineages [41]. In our case the detection of family groups and breeding lines is a desired outcome, which provides deeper insight into the genetic structure of the captive population. We, therefore, present the results obtained by the method described in Pritchard et al. [33]. However, we stopped increasing K when Q values for the next cluster dropped below 0.9 in all individuals as proposed by Schulte et al. [35]. The individuals were assigned to genetic clusters using the highest assignment probability. The dataset was checked for null alleles using Micro-Checker 2.2.3 [42]. We checked all loci for potential linkage using the linkage maps provided by Menotti-Raymond et al. [30], [43]–[45]. A test for linkage disequilibrium was performed in Fstat 2.9.3.2 using a log-likelihood ratio G-statistic with Bonferroni corrections [46]. For the latter test, we removed juveniles from the dataset, as these might erroneously suggest the presence of a linkage disequilibrium due to their close relationships [47].

Measures of genetic differentiation have lately been subject to a broad discussion [48], [49]. Thus, we estimated genetic differentiation between the genetic clusters with three different estimates, D_est_, F_ST_ and R_ST_ using GenAlEx 6.501 [50], [51]. F_ST_ is still a useful measure if the population split is rather recent (e.g. for comparing differentiation among captive populations), while R_ST_ is more useful if the split is deep and if it is likely that mutations have already contributed to population differentiation (e.g. for comparing wildcats and domestic cats) [52], [53]. When we tested D_est_ estimates for correlation with R_ST_ and F_ST_, we found a strong positive correlation between D_est_ and F_ST_ (R^2^ = 0.47, P = 0.004) but a negative correlation with R_ST_ (R^2^ = 0.41, P = 0.01). Therefore, only the results of F_ST_ and R_ST_ are provided. For the analyses of population differentiation, we excluded all individuals which could not clearly be assigned to a cluster at K = 2 based on qi>0.8 and where thus assigned as hybrids (see above; all excluded individuals are marked with * in Table S5 in File S1). Nested AMOVAs based on F_ST_ and R_ST_ were performed in GenAlEx with the sample categories "captive", “domestic” and "wild" as regions and the six genetic clusters as populations.

To analyse the degree of hybridization between domestic cats and wildcats, we performed an analysis in NewHybrids 1.1 Beta 3 [54]. We used Jeffreys-type priors for pi and theta, a burnin of 100,000 sweeps and run 1,000,000 sweeps afterwards. Moreover, we calculated a Principal Component Analysis (PCA) using the adegenet package [55] for R 3.0.3 [56] replacing missing data by the mean frequency of the corresponding allele. For this analysis, we assigned all individuals to their most likely STRUCTURE clusters. Wild-caught individuals that clustered with domestic cats in STRUCTURE as well as captive individuals that clustered with wildcats in STRUCTURE were assigned to a unique cluster. We then simulated 100 first generation hybrids as well as 100 backcrosses with either domestic cats or wildcats and performed a second PCA to inspect the degree of overlap of captive individuals with the simulated hybrids.

For comparing genetic diversity, we calculated the mean number of alleles, allelic richness and inbreeding coefficients (F_IS_) for the captive, domestic and wild samples as well as for the genetic clusters in Fstat. GenAlEx was used to determine the expected (H_e_) and observed (H_o_) heterozygosity for each locus and each population and to test for deviations from Hardy-Weinberg equilibrium (HWE). Furthermore, we tested the number and frequency of private alleles for domestic cats and wildcats (excluding potential hybrids) and analysed the number of alleles shared with captive cats as well as their mean frequencies.

Effective population sizes (N_e_) for each the captive population, domestic population and the wild population (excluding the hybrids) were assessed in ONeSAMP [57], which uses a Bayesian approach. The upper and lower bounds of the prior distribution for N_e_ were 2 and 500, respectively. The captive and wild samples were tested for genetic signatures of population bottlenecks using the Wilcoxon signed rank statistic implemented in Bottleneck 1.2.02 [58]. We examined three different mutational models; the infinite alleles model (IAM), the stepwise mutational model (SMM) and the two-phase mutational model (TPM). However, we mainly considered the TPM, as this is the most likely mutation model for microsatellites [59].

The mean relatedness between individuals within the wild and captive population was calculated using Coancestry [60]. We assessed the performance of all seven relatedness estimators by comparing the results with known relationships and chose the estimator based on Wang [61], which showed the smallest deviation from known relationships and the smallest variance. Additionally, we tested the relatedness between the 19 known breeding pairs of the ex situ population. To measure individual F, four estimators are available in Coancestry. We used the TrioML estimator based on Wang [62] as it fitted our data best based on the relatedness data for the whole captive population (see above).

#### Sequencing

DNA sequences were corrected and aligned by eye as no indels occurred. We excluded ambiguous data from the beginnings and ends of the fragments in the analyses. The final alignment contained 828 bp (positions 12642–13469 based upon GenBank entry FCU20753). Sequences were deposited in GenBank under the accession numbers KM246612-KM246625. The identification of haplotypes was carried out in DnaSP 5 [63]. For calculating a haplotype network we included 198 sequences (Table S4 & S5 in File S1). To verify the assignment of haplotypes to wildcats and domestic cats, we also performed a second analysis including reference samples for European wildcats and domestic cats published by Driscoll et al. [32] (identical haplotypes are given in Table S5 in File S1). The program TCS 1.18 [64] was used to construct a parsimony-based network representation of the mitochondrial haplotypes with a connection limit of 95%.

## Results

### Genetic structure

**Figure 1:**
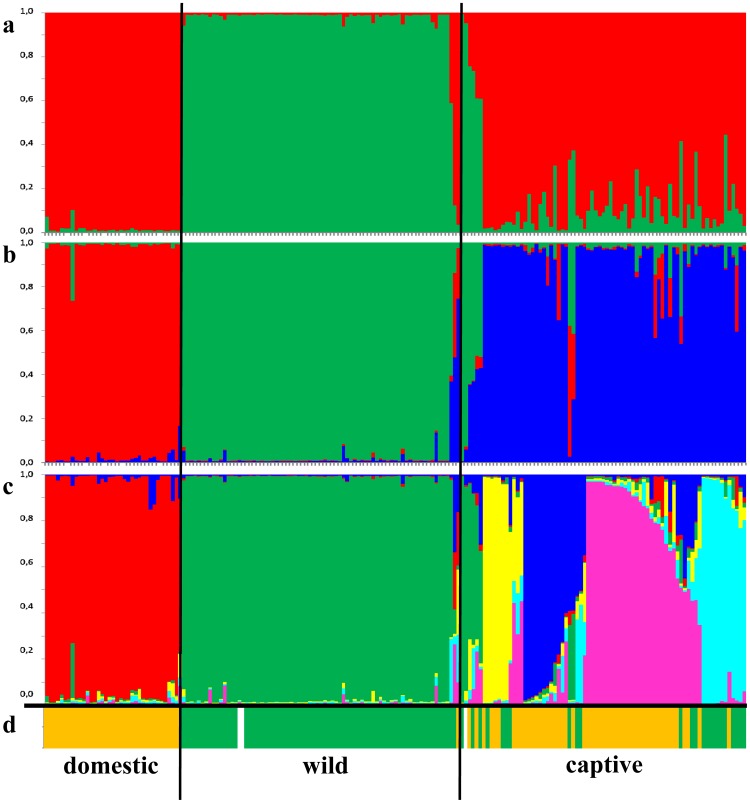
Genetic clusters obtained from the Structure analysis (n = 189). Each individual is represented by a single vertical line, divided into K colours. The coloured segment shows the individual's estimated proportion of membership to that genetic cluster; (a) assignment for K = 2 (red  =  domestic/captive, green  =  wild); (b) assignment for K = 3 (red  =  domestic, green  =  wild, blue  =  captive); (c) assignment for K = 6 (cluster 1: domestic; cluster 2: wild, clusters 3–6: captive). (d) Analogue to the cluster assignments the haplotype affiliation for each individual is indicated by a vertical bar (orange  =  domestic haplotype, green  =  wildcat haplotype, white  =  missing data). For the definition of clusters (geographic origin and ancestry of individuals) see Table 1.

Evidence for null alleles was detected only for single markers in single genetic clusters. All pairwise tests for linkage disequilibrium were non-significant (p>0.05). In the STRUCTURE analysis, we found a clear distinction between individuals from the captive (red cluster in Fig. 1a) and wild (green cluster in Fig. 1a) populations at K = 2. All domestic cats and all wild-caught domestic cats were assigned to the captive cluster (Fig. 1a). At K = 3 (optimal K value according to ΔK; Fig. S3 in File S1) the three groups “wild” (green cluster in Fig. 1b), “captive” (blue cluster in Fig. 1b) and “domestic” (red cluster in Fig. 1b) were clearly distinguished. The most likely number of genetic clusters (K) revealed by Structure was six (Fig. 1c), reflecting geographical groups of breeding lines (Table 1). For example wildcats assigned to cluster 6 (light blue in Fig. 1c) all originated from Scandinavian zoos or had ancestors from these, cluster 3 (yellow in Fig. 1c) comprised mainly ex situ wildcats stemming from one German zoo, and cluster 4 (dark blue in Fig. 1c) mainly included individuals from eastern European zoos. The wild population (green in Fig. 1c) represented a single cluster together with six captive samples. These six samples came mostly from zoos within the range of the wild populations and one of these individuals was a foundling. However not all of them had wildcat mtDNA haplotypes. Another cluster (red in Fig. 1c) included all domestic samples together with the wild-caught domestic cats, some of which were morphologically suspicious.

**Table 1:** Structure based clusters combined with available data on origin and ancestry of the included individuals.

cluster	grouping	individuals lived in or originated from zoos in
cluster 1	domestic/hybrid	all domestic samples and some wild-caught (morphologically suspicious) individuals
cluster 2	wild	wild-caught individuals from the Harz population and single ex situ samples from some German zoos
cluster 3	single German zoo	almost exclusively individuals originating from one German zoo; all four individuals with the Iberian haplotype (WC3) were assigned to this cluster
cluster 4	east-european zoos	cats originating from eastern European zoos or with ancestors from eastern European zoos
cluster 5	various zoos	various zoos
cluster 6	skand. zoos	cats originating from Scandinavian zoos or with ancestors from Scandinavian zoos

Genetic differentiation based upon F_ST_ was significant for all pairs of genetic clusters (p = 0.001, Table 2), but not for R_ST_. The wild cluster (cluster 2) showed a higher differentiation to the captive clusters (mean R_ST_ = 0.032, mean F_ST_ = 0.187) than the domestic cluster (mean R_ST_ = 0.015, mean F_ST_ = 0.163). For the domestic cluster, the highest differentiation occurred to the wild cluster (R_ST_ = 0.059; F_ST_ = 0.254). The nested AMOVA based on F_ST_ revealed that most of the genetic variation occurred within individuals (80%). Yet, a statistically significant portion was explained by the difference between the six genetic clusters (12%) and between the captive, domestic and wild samples (7%). In the R_ST_-based AMOVA, the population level (i.e. the clusters) explained no variation at all, but the three categories (i.e. domestic, wild, captive) explained 7% of the variation. With R_ST_ only 14% of the genetic variation occurred within individuals. The private alleles analysis revealed that captive cats shared 30 private alleles only with domestic cats (mean allele frequency of 21.0%±3.1% SE) and six only with wildcats (8.6%±4.2% SE).

**Table 2:** F_ST_ values (above diagonal) and R_ST_ values (below diagonal) for genetic differentiation between the genetic clusters.

	Cluster 1 (domestic)	Cluster2 (*wild*)	Cluster 3	Cluster 4	Cluster 5	Cluster 6
**Cluster 1**	---	0.254*	0.185*	0.150*	0.147*	0.169*
**Cluster 2**	0.059*	---	0.234*	0.183*	0.147*	0.184*
**Cluster 3**	0.000	0.033	---	0.122*	0.132*	0.181*
**Cluster 4**	0.023	0.019	0.100*	---	0.108*	0.099*
**Cluster 5**	0.036*	0.050*	0.114*	0.161*	---	0.114*
**Cluster 6**	0.000	0.024	0.068*	0.121*	0.074*	---

The in situ samples were assigned to cluster 2, whereas the domestic samples were assigned to cluster 1. Significant values are marked with * (P<0.05).

**Figure 2:**
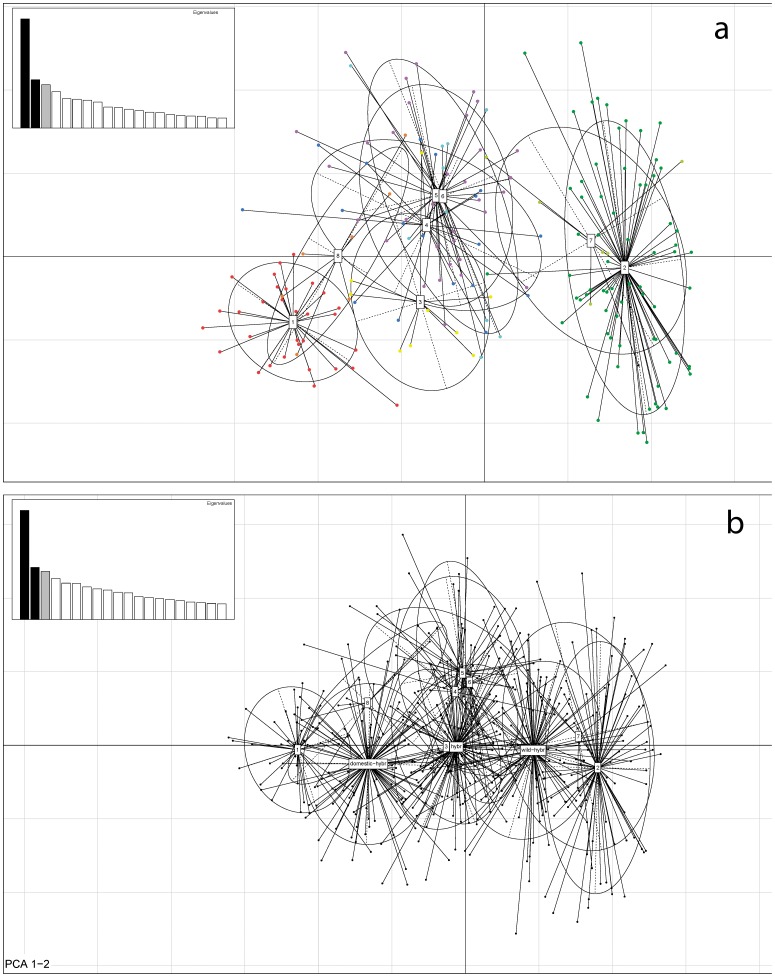
Plot of the first two axes of the Principal component analyses (a) including all obtained genotypes, (b) including simulated first generation hybrids as well as backcrosses with either domestic cats or wildcats. Numbers represent the cluster numbers obtained in STRUCTURE; colours in (a) correspond to the clusters in Figure 1. Captive individuals that were assigned to the wildcat cluster were assigned to a unique group (7) as well as wild-caught individuals that were assigned to the domestic/hybrid cluster (8). The inserted graph shows the distribution of Eigenvalues.

When analysing the data in NewHybrids, only two captive individuals were assigned as purebred wildcats, whereas most captive cats were either assigned as F2 hybrids (62 individuals) or backcrosses with domestic cats (13 individuals). The projected inertia of the PCA was 15.7% for the first axis and 6.9% for the second axis. The first axis separated the domestic cats (negatively loaded, cluster 1 in Fig. 2a) from the wildcats (positively loaded, cluster 2 in Fig. 2a). The captive clusters (3–6) had intermediate positions with negative loadings on the first axis. Captive individuals that clustered with the wildcats in the STRUCTURE analysis (cluster 7 in Fig. 2a) had a strong overlap with the wildcat cluster (except for two individuals). Wild-caught individuals that had been assigned to the domestic/hybrid cluster in STRUCTURE had a strong overlap with the domestic cluster in the PCA as well (cluster 8 in Fig. 2a). When performing the same analysis including the simulated hybrids and back-crosses, there was a substantial overlap of the captive clusters with first generation hybrids (clusters 3–6 in Fig. 2b) and of the wild-caught, morphologically suspicious individuals with second generation backcrosses with domestic cats (cluster 8 in Fig 2b).

**Figure 3:**
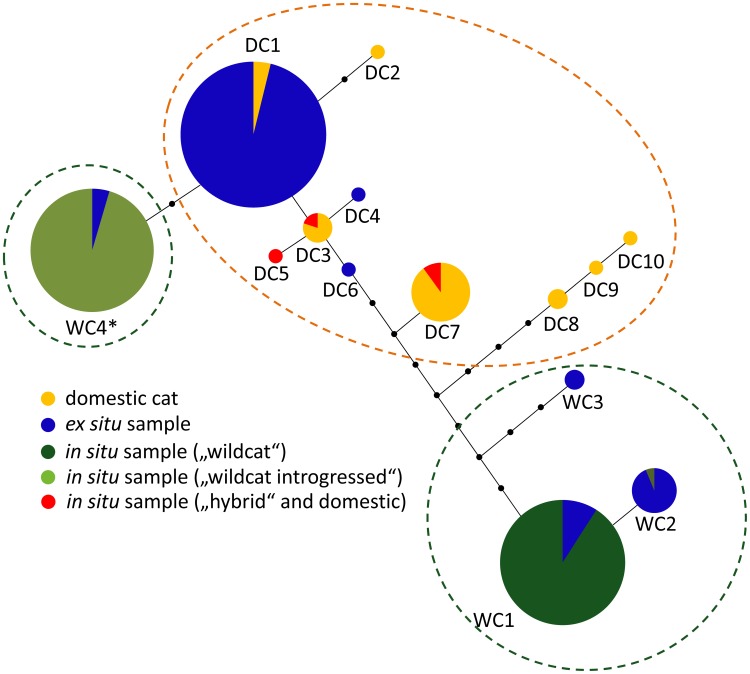
Unrooted parsimony network of mtDNA haplotypes based on an 828 bp sequence of ND5. The sizes of the circles roughly reflect haplotype frequencies in our samples.

We found 14 mtDNA haplotypes in our samples (Fig. 3), two of which (WC1, WC2) were found in 31 in situ wildcats, 15 captive individuals as well as the European wildcat reference samples published by Driscoll et al. [32]. Further ten haplotypes (DC1-DC10) were found in domestic cats (including the domestic cat reference samples), six known or suspected in situ hybrids and 45 zoo samples (as well as GenBank sequences from domestic cats). One haplotype found in four captive individuals (WC3) was identical to a haplotype found in an Iberian wildcat (Fsi257) by Driscoll et al. (2007). The remaining haplotype (WC4*) was found in 36 wild individuals from the Harz population as well as two captive individuals. It was closely related to the most common domestic haplotype DC1 (two substitutions). Altogether 68% (n = 45) of the captive individuals had domestic haplotypes, whereas 32% (n = 21) had haplotypes found in wild populations of *F. s. silvestris* (Figs. 1d, 3). Only one captive cluster (STRUCTURE cluster 6) was little affected by mitochondrial introgression with only a single individual possessing a domestic haplotype. However, all individuals belonging to this cluster were assigned either as F2 hybrids or backcrosses with domestic cats in the NewHybrids analysis.

### Genetic diversity in captive and wild population

The effective population sizes of the captive, wild and domestic populations were 70±1.74, 32±1.11 and 53±3.02 (mean ± SE), respectively. The captive clusters had a higher allelic richness and higher expected and observed heterozygosities than the wild cluster (Table 3). There was little variation in genetic diversity among the captive clusters.

**Table 3:** Genetic diversity and inbreeding in captive, domestic and wild populations and the Structure based clusters.

population/cluster	n	n_a_	A_r_	H_o_	H_e_	F_IS_
cluster 1 (domestic)	32	8.2	5.9	0.731	0.741	0.005
cluster 2 (wild)	79	4.7	3.3	0.539	0.544	−0.012
cluster 3 (captive)	9	3.8	3.8	0.711	0.603	−0.170
cluster 4 (captive)	17	5.4	4.6	0.600	0.691	0.108
cluster 5 (captive)	33	6.3	4.6	0.709	0.697	−0.033
cluster 6 (captive)	12	4.1	3.9	0.717	0.664	−0.138
** mean captive clusters**	**71**	**4.9**	**5.7**	**0.684**	**0.649**	**−0.040**
**captive**	77	7.7	6.9	0.681	0.738	0.085
**domestic**	32	8.2	8.1	0.741	0.741	0.064
**wild**	73	4.1	3.7	0.532	0.533	0.021

n =  sample size; n_a_ =  number of alleles; A_r_ =  allelic richness based on the lowest samples size (n = 9 for the cluster-based analysis; n = 32 for the three sampled populations); H_o_ =  observed heterozygosity; H_e_ =  expected heterozygosity; F_IS_ =  inbreeding coefficient.

The captive population showed significant evidence of a recent bottleneck under both IAM and TPM, but not under SMM. For the domestic population there was only significant evidence of a bottleneck under the IAM, whereas the wild population showed no signs of a bottleneck (Table 4). When the genetic clusters were analysed separately, two captive clusters showed no evidence of a bottleneck, whereas all other clusters showed evidence of a bottleneck under the IAM.

**Table 4:** One-tailed p-values of the Wilcoxon test for heterozygosity excess under three mutational models for captive population, wild population and the genetic clusters.

population/cluster	IAM	TPM	SMM
**captive**	0.00049*	0.00244*	0.98389
**domestic**	0.00488*	0.53906	0.98779
**wild**	0.04199	0.31250	0.98779
**cluster1**	0.00684*	0.57715	0.99316
**cluster2**	0.00684*	0.24609	0.90332
**cluster3**	0.06543	0.24609	0.57715
**cluster4**	0.06543	0.34766	0.91992
**cluster5**	0.00146*	0.27832	0.98389
**cluster6**	0.00244*	0.00488*	0.11621

IAM: infinite alleles model; TPM: two-phase mutational model; SMM: stepwise mutational model. Significant values are marked with * (P<0.005).

The mean relatedness between dyads was r = −0.044±0.007 (mean ± SE) in the wild population and r = −0.042±0.004 in the captive population. The mean individual inbreeding coefficients were 0.149±0.019 in the wild and 0.139±0.019 in the captive population. In both populations most individuals (61%) had an individual inbreeding coefficient <0.125. The number of individuals with an inbreeding coefficient between 0.125 and 0.25 was 18% and 14% for the wild and captive population respectively and the number of individuals with values above 0.25 was 23% and 25%.

Within the breeding pairs we found a mean r of 0.068±0.059. Most breeding pairs (47%) had a low relatedness r≤0.05. However, there were five breeding pairs which showed relatedness values above the half-sib level (0.25) with two of them almost reaching the full sib level (0.5).

## Discussion

### Genetic structure of the European wildcat populations

We found four distinct genetic clusters within the captive population of *F. s. silvestris* (Fig. 1c), which reflected their origin from certain zoos or regional groupings of zoos. The differentiation of the clusters is probably caused by the separation of breeding lines of groups of zoos that often exchanged individuals or zoos that were successful breeders. The existence of isolated breeding lines confirms a lack of knowledge in zoos concerning the stocks of other zoos. Therefore, an exchange of individuals is usually restricted to known partners. When testing the data in STRUCTURE, only six captive individuals were assigned to the wild cluster, some of which were foundlings from the in situ populations that had recently been integrated into the captive population. However, only four of them carried wild mtDNA haplotypes. At K = 2 we found a clear distinction between wild individuals on the one hand and captive individuals together with domestic cats on the other hand. When analysing the data in NewHybrids, only two captive individuals were assigned to purebred wildcats and in the PCA there was a strong overlap of the captive clusters with simulated hybrids and backcrosses. Furthermore, the level of differentiation based upon R_ST_ values was much lower between captive clusters and domestic cats than between captive clusters and wildcats. This suggests that the captive population is strongly influenced by gene flow with domestic cats and that the captive cats are not representative for the wild population.

In the wild population we found two distinct mtDNA clades, which is in agreement with other genetic studies [65], [66]. One mtDNA lineage (consisting of the haplotypes WC1, WC2 and WC3) also included wildcat samples from Rhineland-Palatinate and the Saarland as well as wildcat reference samples of Driscoll et al. [32] (see also table S5 in File S1). This lineage (“wildcat”, Fig. 3) was well separated from the other clades and probably represents the autochthonous mitochondrial lineage of the European wildcat (including the substantially differentiated Iberian clade WC3). The second in situ lineage (“wildcat introgressed”, WC4*, consisting exclusively of individuals from the Harz population and two captive individuals) was closely related to a common domestic cat mtDNA haplotype (DC1). We, therefore, suspect that this lineage originated by ancient introgression from domestic cats. As this mtDNA haplotype is already derived from the domestic cat haplotype and no domestic cat shared exactly the same haplotype, we suppose that introgression probably occurred hundreds to thousands of years ago (due to the relatively small genetic distances a molecular clock is not applicable). This scenario is a reasonable explanation as the wild population in the Harz region has had a long history of geographic isolation due to the extinction of most adjacent populations [26], [65], [66]. During this period of isolation the population has most likely declined due to persecution and some of the remaining wild individuals might well have mated with domestic cats. On the other hand, it remains unknown if this lineage is even more widespread in eastern or south-eastern Europe as samples from this region are scarce. Due to the differences to pure domestic cat haplotypes, we treat this introgressed in situ lineage as a second wildcat lineage, which is also representative for a natural population.

In the wild population we found only few individuals (most of which were roadkills that were known or suspected to be hybrids) who shared haplotypes with domestic cats, confirming that hybridization still occurs in the wild but at a low frequency. Hybridization with domestic cats has been detected in several free-ranging populations of the European wildcat [25], [26], [32], [65]–[69]. In contrast to the past introgression event in the wild population, the captive population seems to be strongly affected by recent introgression, as two thirds of the individuals shared haplotypes with domestic cats (Fig. 1 and 3). We infer, based on the data presented here, that some founders of the captive population were hybrids. Zoos and wildlife sanctuaries are often confronted with stray kittens, which are suspected to be wildcats and are integrated into the captive population (as was the case with some of our sampled individuals). However, there is a high chance that many of these individuals are of hybrid origin. Humans are more likely to encounter litters or stray kittens of domestic cats, as these are tamer and have a higher probability of living close to human settlements [70]. Hybrids are difficult to identify based on their morphology and even the discrimination of some domestic cats from pure wildcats remains difficult [24], [32], [67], [71]. Without genetic screening there remains a high risk of integrating hybrids or backcrosses into the captive population.

### Genetic integrity of the captive population

Our results show that the captive population of the European wildcat represents a hybrid swarm. Only six captive individuals (7.7%) were assigned as wildcats in the STRUCTURE analysis (at K = 2), two of which had domestic cat mtDNA haplotypes, and only two individuals were clearly assigned in the analysis with NewHybrids (one with a domestic cat mtDNA haplotype). Altogether, 68% of the captive population possess domestic cat mtDNA haplotypes. Therefore, the captive population cannot be recommended for further breeding and reintroduction. As mtDNA is maternally inherited, the presence of domestic cat haplotypes in two thirds of the ex situ population does not allow any conclusions on the total amount of domestic cat nuclear introgression into the captive wildcat gene pool. Furthermore, the amount of introgression from male domestic cats remains unknown, as no Y-chromosome markers have been studied. However, the European wildcat is still widespread and it is possible to obtain new founders from the wild. Particularly the large populations in the Hunsrueck Mountains (Rhineland-Palatinate/Saarland) currently show no indication of past introgression, but more detailed studies are required for any final conclusions. Individuals of hybrid origin cannot be recommended for breeding. This leaves many zoos with the difficult decision of what to do with the individuals which do not qualify as "pure" wildcats. One might question whether the introgression affecting the captive population occurred recently or if it reflects another trace of an ancient introgression event. Hertwig et al. [65] suggested that "pure" wildcats without any traces of past introgression do not exist in Central Europe. They hypothesized that repeated introgression from domestic cats, followed by a spread and diversification of the wildcat populations might have played a major role in the establishment of European wildcat populations. However, the domestic cat haplotypes found in the captive population were only found in few wild individuals, all of which were declared to be morphologically suspicious and most probably were wild-caught domestic cats. Although we did not sample the captive individuals ourselves, we occasionally came across some individuals, which were also morphologically suspect. This supports our hypothesis that recent hybrids were integrated into the captive population as founders. Based upon these conclusions we recommend to abstain from any further reintroductions of captive European wildcats from the existing captive stock.

It is very likely that the reintroduced populations of the European wildcat in parts of Germany (e.g. Bavaria, Hesse) represent hybrid swarms as well as they stem from the captive population studied. Removing the reintroduced populations from the wild is not feasible as the administrative effort and costs for such a project would be far too high and it is very unlikely that all individuals could be caught and screened genetically. Moreover, it remains unknown to what extent the reintroduced wildcat populations originated from the captive population or immigrated from wild populations. A genetic survey of the reintroduced populations is therefore needed to assess their status. As the European wildcat currently expands its range (for example in Germany) [22], [65], [72], further reintroduction programmes are not needed and also not cost-efficient. Nevertheless, regional conservation administrations still aim at reintroduction in areas which are currently not colonized naturally.

### Recommendations for a future breeding strategy

Outbreeding is a major threat for the success of *ex situ* conservation programmes [5], [7], [73]. Some principle decisions on the fate of the captive wildcat population have to be made by the holders and zoo associations. The first question concerns the priority of keeping the European wildcat in zoos. Space is a limited resource in zoos and it might be a wise decision to focus on breeding highly endangered species rather than non-threatened ones. However, zoos also have an educational function, which legitimates the display of native wild fauna. Therefore, if the strategic plan of zoo associations comprises a permanent husbandry of European wildcats, large parts of the current stock need to be replaced with pure wildcats. A future captive population should reflect the genetic structure and diversity found in the wild, particularly in populations which are little affected by hybridization with domestic cats. A sporadic integration of wild individuals into the captive breeding stock has been recommended by several authors [74], [75]. In the case of the European wildcat, zoos are in the lucky position that they can obtain individuals from healthy wild populations and, therefore, are able to increase the genetic variability of their breeding stock. Nevertheless, each new individual should be screened genetically before it is integrated into the population.

The current plan to establish a studbook for the European wildcat also requires a coordinated breeding strategy. The most widely accepted management strategy is minimizing kinship [76], which aims at minimizing the overall level of relationship in the population and maximizing the retention of genetic diversity [77], [78]. However, for this approach, detailed pedigree data is needed to find optimal breeding pairs [77], [79]. Genetic analysis during the establishment of a new captive population can help to minimize the risk of inbreeding and outbreeding. An artificial fragmentation of captive populations into several more or less independent subpopulations has been recommended by several authors to retain a maximum of genetic diversity [74], [80]. Although such a fragmentation has been achieved in the current *ex situ* population of *F. s. silvestris* just by chance (due to the missing network of holders), and might have possibly helped to maintain the strong genetic diversity, it is likely that this diversity has mainly been caused by admixture with domestic cats. Increased genetic diversity caused by admixture of several evolutionary lineages has also been reported from invasive non-native populations [35].

### Genetic monitoring of *ex situ* conservation projects

The importance of genetic assessments of captive populations has already been recognized decades ago [81]. Molecular genetic studies of captive populations have provided valuable information for the improvement of conservation breeding programmes [7]. These include the detection of hybrids [82], the detection of genetically valuable individuals that had been excluded from breeding [83], the validation of studbook data and detection of errors [11], [12], and finally the detection of insufficient representation of genetic diversity found in the wild [10].

The present study shows that genetic analyses provide valuable insight into the status of wild and captive populations. Using two different marker systems (microsatellites, mtDNA) helps to unravel introgression patterns. Future studies should also integrate Y-chromosome markers to detect potential male introgression. The integration of hybrids into ex situ populations can have drastic effects on the genetic composition of captive populations. Hybridisation with closely related species or domesticated congeners represents a severe threat to the conservation of endangered species both in situ and ex situ [35], [82], [84]–[87]. Until now, many individuals from the captive population of the European wildcat have been reintroduced to the wild without any test of their genetic integrity. Such reintroductions may strongly deteriorate the genetic integrity of wild populations and promote cryptic extinctions [85]. In some parts of Europe (e.g. Scotland) the wild populations of the wildcat are severely threatened by hybridisation [22]. Therefore, measures to assess and constrict any further admixture of wild and domestic cats are of great importance [22]. Although introgression must have occurred also in nature (perhaps even repeatedly), the European wildcat can still be regarded as a distinct taxon that exists in sympatry with the domestic cat and needs to be managed as a separate unit.

## Supporting Information

File S1 **Text S1**, Permits for sampling. **Text S2**, List of participating institutions for genetic analyses. **Figure S3**, Plots of LnP(D) and delta K (Evanno et al. 2005) for the microsatellite data obtained with STRUCTURE Harvester. **Table S4**, Sampling regime. 1) Most juveniles of sampled mothers and siblings were excluded from sequencing. Their haplotypes were inferred using their maternal relatives (number behind slash). 2) One captive individual was sampled twice and thus the duplicate sample was excluded. 3) The DNA quality of the sample from a preserved captive specimen was not sufficient for sequencing. **Table S5**, Information on the samples used in this study. The table contains all samples which were used in this study, together with their origin, the detected mtDNA haplotypes, the assignments to the STRUCTURE clusters at K = 2, K = 3 and K = 6 as well as the most probable assignment by NewHybrids. At K = 2 all individuals which qualify as hybrids based on qi <0.8 are marked with an *. These individuals were excluded for the analyses of population differentiation. For reasons of data protection the origin of the ex situ samples can only be given as encrypted information. Remarks: 1) DNA quality was too low for genotyping; 2) individual was classified as morphologically suspicious; 3) known hybrid; 4) domestic cat (this individual had a wildcat number as it was included in the wildcat samples as a control by the provider of in situ samples); 5) single samples from wild populations as reference for sequencing, but not suitable for population genetic approaches; 6) zoo located next to wild population in the Harz mountains; 7) foundling within region of the wild population Rhineland Palatinate; 8) the cat was transferred to a new zoo which also took a sample, the duplicate sample was excluded from analysis.
(DOCX)

## References

[1] Butchart S, Walpole M, Collen B, van Strien A, Scharlemann J, et al (2010) Global biodiversity: indicators of recent declines. Science 328: 1164–1168. 20430971 10.1126/science.1187512

[2] Fischer J, Lindenmayer DB (2000) An assessment of the published results of animal relocations. Biol Conserv 96: 1–11.

[3] Storfer A (1999) Gene flow and endangered species translocations: a topic revisited. Biol Conserv 87: 173–180.

[4] WAZA (2005) Building a future for wildlife - The world zoo and aquarium conservation strategy. In: P. Dollinger, editor editors. Bern: WAZA.

[5] Boakes EH, Wang JL (2005) A simulation study on detecting purging of inbreeding depression in captive populations. Genet Res 86: 139–148. 16356287 10.1017/S001667230500772X

[6] Frankham R, Ballou JD, Briscoe DA (2010) Introduction to conservation genetics. Cambridge: University Press. 618 p.

[7] Witzenberger KA, Hochkirch A (2011) Ex situ conservation genetics: a review of molecular studies on the genetic consequences of captive breeding programmes for endangered animal species. Biodivers Conserv 20: 1843–1861.

[8] Cuaron AD (2005) Further role of zoos in conservation: Monitoring wildlife use and the dilemma of receiving donated and confiscated animals. Zoo Biol 24: 115–124.

[9] Marshall TC, Spalton JA (2000) Simultaneous inbreeding and outbreeding depression in reintroduced Arabian oryx. Anim Conserv 3: 241–248.

[10] Tzika AC, Remy C, Gibson R, Milinkovitch MC (2009) Molecular genetic analysis of a captive-breeding program: the vulnerable endemic Jamaican yellow boa. Conserv Genet 10: 69–77.

[11] Bowling AT, Zimmermann W, Ryder O, Penado C, Peto S, et al (2003) Genetic variation in Przewalski's horses, with special focus on the last wild caught mare, 231 Orlitza III. Cytogenet Genome Res 102: 226–234. 14970708 10.1159/000075754

[12] Witzenberger KA, Hochkirch A (2013) Evaluating ex situ conservation projects: Genetic structure of the captive population of the Arabian sand cat. Mamm Biol 78: 379–382.

[13] O'Brien SJ (2006) Animal conservation genetics – an overview with relevance to captive breeding programmes. EAZA News 57: 26–35.

[14] Leberg PL, Firmin BD (2008) Role of inbreeding depression and purging in captive breeding and restoration programmes. Mol Ecol 17: 334–343. 18173505 10.1111/j.1365-294X.2007.03433.x

[15] Hedrick PW, Miller PS, Geffen E, Wayne R (1997) Genetic evaluation of the three captive Mexican wolf lineages. Zoo Biol 16: 47–69.

[16] Henry P, Miquelle D, Sugimoto T, McCullough DR, Caccone A, et al (2009) *In situ* population structure and *ex situ* representation of the endangered Amur tiger. Mol Ecol 18: 3173–3184. 19555412 10.1111/j.1365-294X.2009.04266.x

[17] Boakes EH, Wang JL, Amos W (2007) An investigation of inbreeding depression and purging in captive pedigreed populations. Heredity 98: 172–182. 17180167 10.1038/sj.hdy.6800923

[18] Kraaijeveld-Smit FJL, Griffiths RA, Moore RD, Beebee TJC (2006) Captive breeding and the fitness of reintroduced species: a test of the responses to predators in a threatened amphibian. J Appl Ecol 43: 360–365.

[19] Lau J, Alberts AC, Chemnick LG, Gerber GP, Jones KC, et al (2009) Isolation and characterization of 23 polymorphic microsatellite loci for a West Indian iguana (*Cyclura pinguis*) from the British Virgin Islands. Mol Ecol Res 9: 1412–1414. 10.1111/j.1755-0998.2009.02683.x 21564923

[20] Roldán VA, Navarro JL, Gardenal CN, Martella MB (2010) May captive populations of Greater rhea (*Rhea americana*) act as genetic reservoirs in Argentina? Zoo Biol 29: 1–6. 20235106 10.1002/zoo.20314

[21] Ramirez O, Altet L, Ensenat C, Vilà C, Sanchez A, et al (2006) Genetic assessment of the Iberian wolf *Canis lupus signatus* captive breeding program. Conserv Genet 7: 861–878.

[22] Driscoll C, Nowell K (2010) *Felis silvestris*. 2013 IUCN Red List of Threatened Species. IUCN

[23] Grabe H, Worel G (2001) Die Wildkatze. Zurück auf leisen Pfoten. Amberg: Buch und Kunstverlag Oberpfalz.

[24] Macdonald DW, Yamaguchi N, Kitchener AC, Daniels M, Kilshwa K, et al. (2010) Reversing cryptic extinction: the history, present, and future ot the Scottish wildcat. In: D. W. Macdonald and A. Loveridge, editors. Biology and conservation of wild felids. Oxford, UK: Oxford University Press. pp.471–491.

[25] Lecis R, Pierpaoli M, Birò ZS, Szementhy L, Ragni B, et al (2006) Bayesian analyses of admixture in wild and domestic cats (*Felis silvestris*) using linked microsatellite loci. Mol Ecol 15: 119–131. 16367835 10.1111/j.1365-294X.2005.02812.x

[26] Pierpaoli M, Birò ZS, Herrmann M, Hupe K, Fernandes M, et al (2003) Genetic distinction of wildcat (*Felis silvestris*) populations in Europe, and hybridization with domestic cats in Hungary. Mol Ecol 12: 2585–2598. 12969463 10.1046/j.1365-294x.2003.01939.x

[27] Walsh PS, Metzger DA, Higuchi R (1991) Chelex® 100 as a medium for simple extraction of DNA for PCR-based typing from forensic material. BioTechniques 10: 506–513. 1867860

[28] Estoup A, Largiader CR, Perrot E, Chourrout D (1996) Rapid one-tube DNA extraction for reliable PCR detection of fish polymorphic markers and transgenes. Mol Mar Biol Biotechnol 5: 295–298.

[29] Menotti-Raymond M, O'Brien SJ (1995) Evolutionary conservation of ten microsatellites loci in four species of felidae. The Journal of Heredity 86: 319–322. 7658003 10.1093/oxfordjournals.jhered.a111594

[30] Menotti-Raymond M, David VA, Lyons LA, Schäffer AA, Tomlin JF, et al (1999) A genetic linkage map of microsatellites in the domestic cat (*Felis catus*). Genomics 57: 9–23. 10191079 10.1006/geno.1999.5743

[31] Taberlet P, Griffin S, Goossens B, Questiau S, Manceau V, et al (1996) Reliable genotyping of samples with very low DNA quantities using PCR. Nucleic Acids Res 24: 3189–3194. 8774899 10.1093/nar/24.16.3189 PMC146079

[32] Driscoll CA, Menotti-Raymond M, Roca AL, Hupe K, Johnson WE, et al (2007) The near eastern origin of cat domestication. Science 317: 519–523. 17600185 10.1126/science.1139518 PMC5612713

[33] Pritchard JK, Stephens M, Donnelly P (2000) Inference of population structure using multilocus genotype data. Genetics 155: 945–959. 10835412 10.1093/genetics/155.2.945 PMC1461096

[34] Vähä J-P, Primmer C (2006) Efficiency of model-based Bayesian methods for detecting hybrid individuals under different hybridization scenarios and with different numbers of loci. Mol Ecol 15: 63–72. 16367830 10.1111/j.1365-294X.2005.02773.x

[35] Schulte U, Veith M, Hochkirch A (2012) Rapid genetic assimilation of native wall lizard populations (Podarcis muralis) through extensive hybridization with introduced lineages. Mol Ecol 21: 4313–4326. 22765844 10.1111/j.1365-294X.2012.05693.x

[36] Randi E (2007) Detecting hybridization between wild species and their domesticated relatives. Mol Ecol 17: 285–293. 10.1111/j.1365-294X.2007.03417.x 18173502

[37] Evanno G, Regnaut S, Goudet J (2005) Detecting the number of clusters of individuals using the software STRUCTURE: a simulation study. Mol Ecol 14: 2611–2620. 15969739 10.1111/j.1365-294X.2005.02553.x

[38] Campana MG, Hunt HV, Jones H, White J (2010) CorrSieve: software for summarizing and evaluating Structure output. Mol Ecol Res. 10.1111/j.1755-0998.2010.02917.x 21429142

[39] Hausdorf B, Hennig C (2010) Species delimitation using dominant and codominant multilocus markers. Syst Biol 59: 491–503. 20693311 10.1093/sysbio/syq039

[40] Waples R, Gaggiotti O (2006) What is a population? An empirical evaluation of some genetic methods for identifying the number of gene pools and their degree of connectivity. Mol Ecol 15: 1419–1439. 16629801 10.1111/j.1365-294X.2006.02890.x

[41] Rodrigues ST, Wang J (2012) The effect of close relatives on unsupervised Bayesian clustering algorithms in population genetic structure analysis. Mol Ecol Res 12: 873–884. 10.1111/j.1755-0998.2012.03156.x 22639868

[42] Van Oosterhout C, Hutchinson WF, Wills DPM, Shipley P (2004) MICRO-CHECKER: software for identifying and correcting genotyping errors in microsatellite data. Mol Ecol 4: 535–538.

[43] Menotti-Raymond M, David VA, Chen ZQ, Menotti KA, Sun S, et al (2003) Second-generation integrated genetic linkage/radiation hybrid maps of the domestic cat (*Felis catus*). J Hered 94: 95–106. 12692169 10.1093/jhered/esg008

[44] Menotti-Raymond M, David VA, Schaffer AA, Tomlin JF, Eizirik E, et al (2009) An autosomal genetic linkage map of the domestic cat, *Felis silvestris catus* . Genomics 93: 305–313. 19059333 10.1016/j.ygeno.2008.11.004 PMC2656606

[45] Menotti-Raymond M, David VA, Agarwala R, Schaffer AA, Stephens R, et al (2003) Radiation hybrid mapping of 304 novel microsatellites in the domestic cat genome. Cytogenet Genome Res 102: 272–276. 14970716 10.1159/000075762

[46] Goudet J (1995) FSTAT (Version 1.2): A computer program to calculate F-statistics. J Hered 86: 485–486.

[47] Falush D, Stephens M, Pritchard JK (2003) Inference of population structure using multilocus genotype data: Linked loci and correlated allele frequencies. Genetics 164: 1567–1587. 12930761 10.1093/genetics/164.4.1567 PMC1462648

[48] Jost L (2008) G_ST_ and its relatives do not measure differentiation. Mol Ecol 17: 4015–4026. 19238703 10.1111/j.1365-294x.2008.03887.x

[49] Gerlach G, Jueterbock A, Kraemer P, Deppermann J, Harmand P (2010) Calculations of population differentiation based on G_ST_ and D: forget G_ST_ but not all of statistics!. Mol Ecol 19: 3845–3852. 20735737 10.1111/j.1365-294X.2010.04784.x

[50] Peakall R, Smouse PE (2006) GENALEX 6: genetic analysis in Excel. Population genetic software for teaching and research. Mol Ecol Res 6: 288–295. 10.1093/bioinformatics/bts460 PMC3463245 22820204

[51] Peakall R, Smouse PE (2012) GenAlEx 6.5: genetic analysis in Excel. Population genetic software for teaching and research—an update. Bioinformatics 28: 2537–2539. 22820204 10.1093/bioinformatics/bts460 PMC3463245

[52] Slatkin M (1995) A measure of population subdivision based on microsatellite allele frequencies. Genetics 139: 457–462. 7705646 10.1093/genetics/139.1.457 PMC1206343

[53] Lugon-Moulin N, Brünner H, Wyttenbach A, Hausser J, Goudet J (1999) Hierarchical analyses of genetic differentiation in a hybrid zone of *Sorex araneus* (Insectivora: Soricidae). Mol Ecol 8: 419–431.

[54] Anderson EC, Thompson EA (2002) A model-based method for identifying species hybrids using multilocus genetic data. Genetics 160: 1217–1229. 11901135 10.1093/genetics/160.3.1217 PMC1462008

[55] Jombart T (2008) adegenet: a R packae fort he multivariate analysis of genetic markers. Bioinformatics 24: 1403–1405. 18397895 10.1093/bioinformatics/btn129

[56] R Development Core Team (2014) R: A Language and environment for statistical computing. Vienna: R Foundation for Statistical Computing.

[57] Tallmon DA, Koyuk A, Luikart G, Beaumont MA (2008) ONeSAMP: a program to estimate effective population size using approximate Bayesian computation. Mol Ecol Res 8: 299–301. 10.1111/j.1471-8286.2007.01997.x 21585773

[58] Cornuet JM, Luikart G (1996) Description and power analysis of two tests for detecting recent population bottlenecks from allele frequency data. Genetis 144: 2001–2014. 10.1093/genetics/144.4.2001 PMC1207747 8978083

[59] Piry S, Luikart G, Cornuet J (1999) Computer note. BOTTLENECK: a computer program for detecting recent reductions in the effective size using allele frequency data. J Hered 90: 502–503.

[60] Wang J (2010) COANCESTRY: a program for simulating, estimating and analysing relatedness and inbreeding coefficients. Mol Ecol Res 11: 141–145. 10.1111/j.1755-0998.2010.02885.x 21429111

[61] Wang JL (2002) An estimator for pairwise relatedness using molecular markers. Genetics 160: 1203–1215. 11901134 10.1093/genetics/160.3.1203 PMC1462003

[62] Wang J (2007) Triadic IBD coefficients and applications to estimating pairwise relatedness. Genet Res 89: 135–153. 17894908 10.1017/S0016672307008798

[63] Librado P, Rozas J (2009) DnaSP v5: a software for comprehensive analysis of DNA polymorphism data. Bioinformatics 25: 1451–1452. 19346325 10.1093/bioinformatics/btp187

[64] Clement M, Posada D, Crandall KA (2000) TCS: a computer program to estimate gene genealogies. Mol Ecol 9: 1657–1659. 11050560 10.1046/j.1365-294x.2000.01020.x

[65] Hertwig ST, Schweizer M, Stepanow S, Jungnickel A, Böhle U-R, et al (2009) Regionally high rates of hybridization and introgression in German wildcat populations (*Felis silvestris*, Carnivora, Felidae). J Zool Syst Evol Res 47: 283–297.

[66] Eckert I, Suchentrunk F, Markov G, Hartl GB (2010) Genetic diversity and integrity of German wildcat (*Felis silvestris*) populations as revealed by microsatellites, allozymes, and mitochondrial DNA sequences. Mamm Biol 75: 160–174.

[67] Randi E, Pierpaoli M, Beaumont MA, Ragni B, Sforzi A (2001) Genetic identification of wild and domestic cats (*Felis silvestris*) and their hybrids using bayesian clustering methods. Mol Biol Evol 18: 1679–1693. 11504848 10.1093/oxfordjournals.molbev.a003956

[68] O'Brien J, Devillard S, Say L, Vanthomme H, Leger F, et al (2009) Preserving genetic integrity in a hybridising world: are European Wildcats (*Felis silvestris silvestris*) in eastern France distinct from sympatric feral domestic cats? Biodivers Conserv 18: 2351–2360.

[69] Oliveira R, Godinho R, Randi E, Alves PC (2008) Hybridization versus conservation: are domestic cats threatening the genetic integrity of wildcats (*Felis silvestris silvestris*) in Iberian Peninsula? Philosophical Transactions of the Royal Society B-Biological Sciences 363: 2953–2961. 10.1098/rstb.2008.0052 PMC2606743 18522917

[70] Germain E, Benhamou S, Poulle ML (2008) Spatio-temporal sharing between the European wildcat, the domestic cat and their hybrids. J Zool 276: 195–203.

[71] Hubbard AL, McOrist S, Jones TW, Boid R, Scott R, et al (1992) Is survival of European wildcats Felis silvestris in Britain threatened by interbreeding with domestic cats? Biol Conserv 61: 203–208.

[72] Steyer K, Simon O, Kraus RHS, Haase P, Nowak C (2013) Hair trapping with valerian-treated lure sticks as a tool for genetic wildcat monitoring in low-density habitats. Eur J Wildl Res 59: 39–46.

[73] Frankham R (2010) Challenges and opportunities of genetic approaches to biological conservation. Biol Conserv 143: 1919–1927.

[74] Frankham R (2008) Genetic adaptation to captivity in species conservation programs. Mol Ecol 17: 325–333. 18173504 10.1111/j.1365-294X.2007.03399.x

[75] Williams SE, Hoffman EA (2009) Minimizing genetic adaptation in captive breeding programs: A review. Biol Conserv 142: 2388–2400.

[76] Falconer DS, Mackay TFC (1996) Introduction to quantitative genetics. Harlow, England: Longman.

[77] Ballou JD, Lacy RC (1995) Identifying genetically important individuals for management of genetic diversity in pedigreed populations. In: J. DBallou, MGilpin and T. JFoose, editors. Population Management for Survival & Recovery Analytical Methods and Strategies in Small Population Conservation. New York: Columbia University Press. pp. 76–111.

[78] Saura M, Perez-Figueroa A, Fernandez J, Toro MA, Caballero A (2008) Preserving population allele frequencies in *ex situ* conservation programs. Conserv Biol 22: 1277–1287. 18680505 10.1111/j.1523-1739.2008.00992.x

[79] Caballero A, Toro MA (2000) Interrelations between effective population size and other pedigree tools for the management of conserved populations. Genet Res 75: 331–343. 10893869 10.1017/s0016672399004449

[80] Robert A (2009) Captive breeding genetics and reintroduction success. Biol Conserv 142: 2915–2922.

[81] Templeton AR, Davis SK, Read B (1987) Genetic variability in a captive herd of Speke's gazelle (*Gazella spekei*). Zoo Biol 6: 305–313.

[82] Luo S-J, Johnson WE, Martenson J, Antunes A, Martelli P, et al (2008) Subspecies genetic assignments of worldwide captive tigers increase conservation value of captive populations. Curr Biol 18: 592–596. 18424146 10.1016/j.cub.2008.03.053

[83] Goncalves da Silva A, Lalonde DR, Quse V, Shoemaker A, Russello MA (2010) Genetic approaches refine *ex situ* Lowland Tapir (*Tapirus terrestris*) conservation. J Hered 101: 581–590. 20484384 10.1093/jhered/esq055

[84] Gottelli D, Sillerozubiri C, Applebaum GD, Roy MS, Girman DJ, et al (1994) Molecular-genetics of the most endangered canid - the Ethiopian wolf (*Canis simensis*). Mol Ecol 3: 301–312. 7921357 10.1111/j.1365-294x.1994.tb00070.x

[85] Rhymer J, Simberloff D (1996) Extinction by hybridization and introgression. Annu Rev Ecol Syst 27: 83–109.

[86] Allendorf FW, Leary RF, Spruell P, Wenburg JK (2001) The problems with hybrids: setting conservation guidelines. Trends Ecol Evol 16: 613–622.

[87] Ely JJ, Dye B, Frels WI, Fritz J, Gagneux P, et al (2005) Subspecies composition and founder contribution of the captive US chimpanzee (*Pan troglodytes*) population. Am J Primatol 67: 223–241. 16229023 10.1002/ajp.20179

