# Supplementary material for: The Genetic Integrity of the Ex Situ Population of the European Wildcat (Felis silvestris silvestris) Is Seriously Threatened by Introgression from Domestic Cats (Felis silvestris catus)
Source: PLoS One. 2014 Aug 27;9(8):e106083. doi: 10.1371/journal.pone.0106083 (PMC4146591; doi:10.1371/journal.pone.0106083)
Supplement: File S1 — Text S1, Permits for sampling. Text S2, List of participating institutions for genetic analyses. Figure S3, Plots of LnP(D) and delta K (Evanno et al. 2005) for the microsatellite data obtained with STRUCTURE Harvester. Table S4, Sampling regime. 1) Most juveniles of sampled mothers and siblings were excluded from sequencing. Their haplotypes were inferred using their maternal relatives (number behind slash). 2) One captive individual was sampled twice and thus the duplicate sample was excluded. 3) The DNA quality of the sample from a preserved captive specimen was not sufficient for sequencing. Table S5, Information on the samples used in this study. The table contains all samples which were used in this study, together with their origin, the detected mtDNA haplotypes, the assignments to the STRUCTURE clusters at K = 2, K = 3 and K = 6 as well as the most probable assignment by NewHybrids. At K = 2 all individuals which qualify as hybrids based on qi <0.8 are marked with an *. These individuals were excluded for the analyses of population differentiation. For reasons of data protection the origin of the ex situ samples can only be given as encrypted information. Remarks: 1) DNA quality was too low for genotyping; 2) individual was classified as morphologically suspicious; 3) known hybrid; 4) domestic cat (this individual had a wildcat number as it was included in the wildcat samples as a control by the provider of in situ samples); 5) single samples from wild populations as reference for sequencing, but not suitable for population genetic approaches; 6) zoo located next to wild population in the Harz mountains; 7) foundling within region of the wild population Rhineland Palatinate; 8) the cat was transferred to a new zoo which also took a sample, the duplicate sample was excluded from analysis. (DOCX) [file pone.0106083.s001.docx]

**Supplementary Material**

**Text S1.** Permits for sampling.

In situ samples were taken with the following permits:

Animal welfare permit: „Anzeige eines Tierversuches im Biosphärenreservat Karstlandschaft Südharz (42502-3-411 BioRes)“ from 30 January 2004 via the commissioner for animal welfar, Dr. H. Piegert

Species conservation permit by the Landesverwaltungsamt Sachsen-Anhalt, Mrs. Israel (Permit no. 407.5.1_128/04).

Both permits included the collection of samples from European Wildcats.

**Text S2.** List of participating institutions for genetic analyses.

Alpenzoo Innsbruck, Heimat- Tierpark Olderdissen Bielefeld, Jardin Zoologico y botanica "Alberto Duran" (Jerez de la Frontera), Le Parc Des Felins, Zoo Gdansk, Novosibirsk Zoo, Opelzoo Kronberg, Cracow Zoo, Poznań Zoo, Skanes Djurpark, Tierfreigehege im Nationalpark Bayrischer Wald, Tiergarten Wels, Tierpark Chemnitz, Tierpark Berlin, Tierpark der Fontanestadt Neuruppin Kunsterspring, Tierpark Hexentanzplatz Thale, Tierpark Nordhorn, Tierpark Petersberg, Wiesentgehege Springe, Wildfreigehege Wildenburg, Wildpark "Alte Fasanerie" Klein-Auheim, Wildpark Bad Mergentheim, Wildpark Frankenhof, Wildpark Grafenberger Wald, Wildpark Klaushof, Wildgehege Moritzburg, Wildpark Pforzheim, Wildpark Reuschenberg, Wildpark Schloß Tambach, Wildpark Tiergarten Weilburg and the Zoo Neunkirchen.

**Figure S3.** Plots of LnP(D) and delta K (Evanno et al. 2005) for the microsatellite data obtained with STRUCTURE Harvester.

**Table S4.** Sampling regime. 1) Most juveniles of sampled mothers and siblings were excluded from sequencing. Their haplotypes were inferred using their maternal relatives (number behind slash). 2) One captive individual was sampled twice and thus the duplicate sample was excluded. 3) The DNA quality of the sample from a preserved captive specimen was not sufficient for sequencing.

| **sample category** | **origin** | **n samples** | **n samples with sufficient DNA quality** | **n samples used for microsatellite analysis** | **n samples used for mtDNA sequencing ^1)^** |
| --- | --- | --- | --- | --- | --- |
| captive | ex situ population | 80 | 78 | 77 ^2)^ | 66 / 77 |
| wild | wild population Harz mountains | 89 | 84 | 79 | 64 ^3)^ / 82 |
| wild | wild-caught individuals from Rhineland-Palatinate (2) and Saarland (4) | 6 | 6 | 0 | 6 / 6 |
| domestic | domestic cats | 33 | 33 | 33 | 33 / 33 |

**Table S5.** Information on the samples used in this study.

The table contains all samples which were used in this study, together with their origin, the detected mtDNA haplotypes, the assignments to the STRUCTURE clusters at K=2, K=3 and K=6 as well as the most probable assignment by NewHybrids. At K = 2 all individuals which qualify as hybrids based on qi < 0.8 are marked with an *. These individuals were excluded for the analyses of population differentiation. For reasons of data protection the origin of the ex situ samples can only be given as encrypted information. Remarks: 1) DNA quality was too low for genotyping; 2) individual was classified as morphologically suspicious; 3) known hybrid; 4) domestic cat (this individual had a wildcat number as it was included in the wildcat samples as a control by the provider of in situ samples); 5) single samples from wild populations as reference for sequencing, but not suitable for population genetic approaches; 6) zoo located next to wild population in the Harz mountains; 7) foundling within region of the wild population Rhineland Palatinate; 8) the cat was transferred to a new zoo which also took a sample, the duplicate sample was excluded from analysis

| **sample ID** | **sample group** | **origin** | **sex** | **sample type** | **mtDNA haplotype** | **Driscoll haplotype** | **STRUCTURE assignment** (according to highest Q value) | | | **NewHybrids assignment** | **genotype available** | **remark** |
| --- | --- | --- | --- | --- | --- | --- | --- | --- | --- | --- | --- | --- |
|  |  |  |  |  |  |  | **K = 2** | **K = 3** | **K = 6** |  |  |  |
| WK001 | wild | Harz population (M. Götz) | f | hair/tissue | WC4* | Fsi215 | wild | wild | 2 | Wildcat | yes |  |
| WK003 | wild | Harz population (M. Götz) | f | hair | WC4* | Fsi215 | wild | wild | 2 | Wildcat | yes |  |
| WK005 | wild | Harz population (M. Götz) | f | hair | WC4* | Fsi215 | wild | wild | 2 | Wildcat | yes |  |
| WK006 | wild | Harz population (M. Götz) | m | hair | WC4* | Fsi215 | wild | wild | 2 | Wildcat | yes |  |
| WK007 | wild | Harz population (M. Götz) | m | hair | WC4* | Fsi215 | wild | wild | 2 | Wildcat | yes |  |
| WK008 | wild | Harz population (M. Götz) | m | hair | WC4* | Fsi215 | wild | wild | 2 | Wildcat | yes |  |
| WK009 | wild | Harz population (M. Götz) | f | hair | WC4* | Fsi215 | na | na | na | na | na | 1) |
| WK010 | wild | Harz population (M. Götz) | m | hair | WC4* | Fsi215 | wild | wild | 2 | Wildcat | yes |  |
| WK011 | wild | Harz population (M. Götz) | m | hair/tissue | WC4* | Fsi215 | wild | wild | 2 | Wildcat | yes |  |
| WK012 | wild | Harz population (M. Götz) | m | hair | WC4* | Fsi215 | wild | wild | 2 | Wildcat | yes |  |
| WK014 | wild | Harz population (M. Götz) | m | hair/tissue | WC4* | Fsi215 | wild | wild | 2 | Wildcat | yes |  |
| WK015 | wild | Harz population (M. Götz) | m | hair | WC4* | Fsi215 | wild | wild | 2 | Wildcat | yes |  |
| WK016 | wild | Harz population (M. Götz) | m | hair/tissue | WC4* | Fsi215 | wild | wild | 2 | Wildcat | yes |  |
| WK017 | wild | Harz population (M. Götz) | m | hair | WC4* | Fsi215 | domestic/captive | captive | 4 | F2 Hybrid | yes | 2) |
| WK018 | wild | Harz population (M. Götz) | m | hair | WC1 | Fsi246 | wild | wild | 2 | Wildcat | yes |  |
| WK019 | wild | Harz population (M. Götz) | m | hair/tissue | WC4* | Fsi215 | wild | wild | 2 | Wildcat | yes |  |
| WK020 | wild | Harz population (M. Götz) | m | hair | WC1 | Fsi246 | wild | wild | 2 | Wildcat | yes |  |
| WK021 | wild | Harz population (M. Götz) | m | hair | na | na | wild | wild | 2 | Wildcat | yes |  |
| WK022 | wild | Harz population (M. Götz) | m | hair | na | na | wild | wild | 2 | Wildcat | yes |  |
| WK023 | wild | Harz population (M. Götz) | f | hair | WC4* | Fsi215 | na | na | na | na | na | 1) |
| WK024 | wild | Harz population (M. Götz) | f | hair | WC4* | Fsi215 | wild | wild | 2 | Wildcat | yes |  |
| WK025 | wild | Harz population (M. Götz) | f | hair | WC4* | Fsi215 | wild | wild | 2 | Wildcat | yes |  |
| WK026 | wild | Harz population (M. Götz) | m | hair | WC4* | Fsi215 | wild | wild | 2 | Wildcat | yes |  |
| WK027 | wild | Harz population (M. Götz) | m | hair | WC4* | Fsi215 | wild | wild | 2 | Wildcat | yes |  |
| WK028 | wild | Harz population (M. Götz) | m | hair | WC4* | Fsi215 | wild | wild | 2 | Wildcat | yes |  |
| WK029 | wild | Harz population (M. Götz) | m | hair | WC4* | Fsi215 | wild | wild | 2 | Wildcat | yes |  |
| WK030 | wild | Harz population (M. Götz) | f | hair | WC1 | Fsi246 | wild | wild | 2 | Wildcat | yes |  |
| WK031 | wild | Harz population (M. Götz) | m | hair | WC1 | Fsi246 | wild | wild | 2 | Wildcat | yes |  |
| WK032 | wild | Harz population (M. Götz) | f | hair | WC1 | Fsi246 | wild | wild | 2 | Wildcat | yes |  |
| WK033 | wild | Harz population (M. Götz) | m | hair | WC1 | Fsi246 | wild | wild | 2 | Wildcat | yes |  |
| WK034 | wild | Harz population (M. Götz) | f | hair | WC1 | Fsi246 | wild | wild | 2 | Wildcat | yes |  |
| WK035 | wild | Harz population (M. Götz) | m | hair | WC1 | Fsi246 | wild | wild | 2 | Wildcat | yes |  |
| WK036 | wild | Harz population (M. Götz) | m | hair | WC1 | Fsi246 | wild | wild | 2 | Wildcat | yes |  |
| WK037 | wild | Harz population (M. Götz) | m | hair | WC1 | Fsi246 | wild | wild | 2 | Wildcat | yes |  |
| WK038 | wild | Harz population (M. Götz) | f | hair | WC1 | Fsi246 | na | na | na | na | na | 1) |
| WK039 | wild | Harz population (M. Götz) | f | hair | WC1 | Fsi246 | wild | wild | 2 | Wildcat | yes |  |
| WK040 | wild | Harz population (M. Götz) | f | hair | WC1 | Fsi246 | wild | wild | 2 | Wildcat | yes |  |
| WK041 | wild | Harz population (M. Götz) | f | hair | WC1 | Fsi246 | wild | wild | 2 | Wildcat | yes |  |
| WK042 | wild | Harz population (M. Götz) | m | hair | WC1 | Fsi246 | wild | wild | 2 | Wildcat | yes |  |
| WK043 | wild | Harz population (M. Götz) | m | hair/tissue | WC1 | Fsi246 | wild | wild | 2 | Wildcat | yes |  |
| WK044 | wild | Harz population (M. Götz) | f | hair | WC1 | Fsi246 | wild | wild | 2 | Wildcat | yes |  |
| WK045 | wild | Harz population (M. Götz) | f | hair | WC1 | Fsi246 | wild | wild | 2 | Wildcat | yes |  |
| WK046 | wild | Harz population (M. Götz) | f | hair | WC1 | Fsi246 | wild | wild | 2 | Wildcat | yes |  |
| WK047 | wild | Harz population (M. Götz) | f | hair | WC1 | Fsi246 | na | na | na | na | na | 1) |
| WK048 | wild | Harz population (M. Götz) | f | hair | WC1 | Fsi246 | na | na | na | na | na | 1) |
| WK049 | wild | Harz population (M. Götz) | f | hair | WC4* | Fsi215 | wild | wild | 2 | Wildcat | yes |  |
| WK050 | wild | Harz population (M. Götz) | m | hair | WC4* | Fsi215 | wild | wild | 2 | Wildcat | yes |  |
| WK051 | wild | Harz population (M. Götz) | f | hair | WC4* | Fsi215 | wild | wild | 2 | Wildcat | yes |  |
| WK052 | wild | Harz population (M. Götz) | f | hair | WC4* | Fsi215 | wild | wild | 2 | Wildcat | yes |  |
| WK053 | wild | Harz population (M. Götz) | f | hair | WC4* | Fsi215 | wild | wild | 2 | Wildcat | yes |  |
| WK054 | wild | Harz population (M. Götz) | f | hair | WC4* | Fsi215 | wild | wild | 2 | Wildcat | yes |  |
| WK055 | wild | Harz population (M. Götz) | f | hair | WC4* | Fsi215 | wild | wild | 2 | Wildcat | yes |  |
| WK056 | wild | Harz population (M. Götz) | m | hair | WC4* | Fsi215 | wild | wild | 2 | Wildcat | yes |  |
| WK057 | wild | Harz population (M. Götz) | m | hair | WC1 | Fsi246 | wild | wild | 2 | Wildcat | yes |  |
| WK058 | wild | Harz population (M. Götz) | m | hair | WC4* | Fsi215 | wild | wild | 2 | Wildcat | yes |  |
| WK059 | wild | Harz population (M. Götz) | m | hair | WC1 | Fsi246 | wild | wild | 2 | Wildcat | yes |  |
| WK060 | wild | Harz population (M. Götz) | m | hair | WC4* | Fsi215 | wild | wild | 2 | Wildcat | yes |  |
| WK061 | wild | Harz population (M. Götz) | m | tissue | WC4* | Fsi215 | wild | wild | 2 | Wildcat | yes |  |
| WK062 | wild | Harz population (M. Götz) | ? | tissue | WC1 | Fsi246 | wild | wild | 2 | Wildcat | yes |  |
| WK063 | wild | Harz population (M. Götz) | ? | tissue | DC5 | Fca2718 | domestic/captive | captive | 3 | Backcross Domestic | yes | 2) |
| WK064 | wild | Harz population (M. Götz) | ? | tissue | DC7 | Fca1914c | domestic/captive | domestic | 1 | Domestic | yes |  |
| WK065 | wild | Harz population (M. Götz) | ? | tissue | WC1 | Fsi246 | wild | wild | 2 | Wildcat | yes |  |
| WK066 | wild | Harz population (M. Götz) | ? | tissue | WC1 | Fsi246 | wild | wild | 2 | Wildcat | yes |  |
| WK068 | wild | Harz population (M. Götz) | ? | tissue | WC1 | Fsi246 | wild | wild | 2 | Wildcat | yes |  |
| WK069 | wild | Harz population (M. Götz) | ? | tissue | WC4* | Fsi215 | wild | wild | 2 | Wildcat | yes |  |
| WK070 | wild | Harz population (M. Götz) | ? | tissue | WC1 | Fsi246 | wild | wild | 2 | Wildcat | yes |  |
| WK071 | wild | Harz population (M. Götz) | ? | tissue | WC4* | Fsi215 | wild | wild | 2 | Wildcat | yes |  |
| WK072 | wild | Harz population (M. Götz) | ? | tissue | WC1 | Fsi246 | wild | wild | 2 | Wildcat | yes |  |
| WK074 | wild | Harz population (M. Götz) | ? | tissue | WC1 | Fsi246 | wild | wild | 2 | Wildcat | yes |  |
| WK075 | wild | Harz population (M. Götz) | ? | tissue | WC1 | Fsi246 | wild | wild | 2 | Wildcat | yes |  |
| WK076 | wild | Harz population (M. Götz) | ? | tissue | WC4* | Fsi215 | wild | wild | 2 | Wildcat | yes |  |
| WK077 | wild | Harz population (M. Götz) | ? | tissue | WC4* | Fsi215 | wild | wild | 2 | Wildcat | yes |  |
| WK078 | wild | Harz population (M. Götz) | ? | tissue | DC7 | Fca1914c | domestic/captive | domestic | 1 | Backcross Domestic | yes |  |
| WK079 | wild | Harz population (M. Götz) | ? | tissue | DC3 | Fca2700 | domestic/captive | domestic | 1 | Backcross Domestic | yes | 2) |
| WK080 | wild | Harz population (M. Götz) | ? | tissue | WC1 | Fsi246 | wild | wild | 2 | Wildcat | yes |  |
| WK081 | wild | Harz population (M. Götz) | ? | tissue | WC4* | Fsi215 | wild | wild | 2 | Wildcat | yes |  |
| WK083 | wild | Harz population (M. Götz) | ? | hair | DC3 | Fca2700 | domestic/captive | domestic | 1 | Domestic | yes | 3) |
| WK084 | domestic | Harz population (M. Götz) | ? | hair | DC7 | Fca1914c | domestic/captive | domestic | 1 | Domestic | yes | 4) |
| WK085 | wild | Harz population (M. Götz) | m | tissue | WC4* | Fsi215 | wild | wild | 2 | Wildcat | yes |  |
| WK086 | wild | Harz population (M. Götz) | ? | tissue | WC4* | Fsi215 | wild | wild | 2 | Wildcat | yes |  |
| WK087 | wild | Harz population (M. Götz) | f | tissue | WC1 | Fsi246 | wild | wild | 2 | Wildcat | yes |  |
| WK088 | wild | Harz population (M. Götz) | f | tissue | WC4* | Fsi215 | wild | wild | 2 | Wildcat | yes |  |
| WK089 | wild | Harz population (M. Götz) | m | tissue | WC1 | Fsi246 | wild | wild | 2 | Wildcat | yes |  |
| WK090 | wild | Harz population (M. Götz) | f | tissue | WC1 | Fsi246 | wild | wild | 2 | Wildcat | yes |  |
| WK091 | wild | Harz population (M. Götz) | m | tissue | WC4* | Fsi215 | wild * | wild | 2 | Wildcat | yes |  |
| WK092_2 | wild | Saarland (Zentrum für Biodokumentation) | ? | tissue | WC2 | Fsi26 | na | na | na | na | na | 5) |
| WK093_2 | wild | Saarland (Zentrum für Biodokumentation) | ? | tissue | WC1 | Fsi246 | na | na | na | na | na | 5) |
| WK095 | wild | Saarland (K. Diergarten) | m | hair | WC1 | Fsi246 | na | na | na | na | na | 5) |
| WK096 | wild | Rhineland-Palatinate, Böschfeld |  | hair | WC1 | Fsi246 | na | na | na | na | na | 5) |
| WK097 | wild | Saarland (K. Diergarten) | f | hair | WC1 | Fsi246 | na | na | na | na | na | 5) |
| WK098 | wild | Rhineland-Palatinate, Hunsrück | ? | tissue | WC1 | Fsi246 | na | na | na | na | na | 5) |
| FSS001 | captive | zoo 14 | f | hair | DC1 | Fca1047A | domestic/captive | captive | 5 | F2 Hybrid | yes |  |
| FSS002 | captive | zoo 14 | m | hair | DC1 | Fca1047A | domestic/captive | captive | 5 | F2 Hybrid | yes |  |
| FSS003 | captive | zoo 13 | f | buccal swap | WC4* | Fsi215 | wild | wild | 2 | Wildcat | yes | 6) |
| FSS004 | captive | zoo 13 | m | buccal swap | DC1 | Fca1047A | domestic/captive | captive | 5 | F2 Hybrid | yes |  |
| FSS005 | captive | zoo 13 | m | buccal swap | DC1 | Fca1047A | domestic/captive | captive | 5 | Backcross Domestic | yes |  |
| FSS006 | captive | zoo 13 | f | buccal swap | DC1 | Fca1047A | domestic/captive | captive | 4 | Backcross Domestic | yes |  |
| FSS007 | captive | zoo 13 | m | buccal swap | DC1 | Fca1047A | domestic/captive | captive | 5 | F2 Hybrid | yes |  |
| FSS008 | captive | zoo 13 | f | buccal swap | WC4* | Fsi215 | wild * | wild | 2 | F2 Hybrid | yes | 6) |
| FSS009 | captive | zoo 7 | m | hair | DC1 | Fca1047A | domestic/captive | captive | 5 | F2 Hybrid | yes |  |
| FSS010 | captive | zoo 7 | m | hair | DC1 | Fca1047A | domestic/captive | captive | 5 | F2 Hybrid | yes |  |
| FSS011 | captive | zoo 7 | f | hair | DC1 | Fca1047A | domestic/captive | captive | 5 | F2 Hybrid | yes |  |
| FSS013 | captive | zoo 7 | f | hair | DC1 | Fca1047A | domestic/captive | captive | 5 | F2 Hybrid | yes |  |
| FSS014 | captive | zoo 8 | m | hair | DC1 | Fca1047A | domestic/captive | captive | 6 | F2 Hybrid | yes |  |
| FSS015 | captive | zoo 25 | f | tissue and hair | DC1 | Fca1047A | domestic/captive | captive | 5 | F2 Hybrid | yes |  |
| FSS016 | captive | zoo 17 | f | hair | WC3 | Fsi257 | domestic/captive | captive | 3 | F2 Hybrid | yes |  |
| FSS017 | captive | zoo 15 | f | hair | WC3 | Fsi257 | domestic/captive | captive | 3 | F2 Hybrid | yes |  |
| FSS018 | captive | zoo 15 | m | hair | WC2 | Fsi26 | domestic/captive* | captive | 5 | F2 Hybrid | yes |  |
| FSS019 | captive | zoo 12 | m | hair | WC2 | Fsi26 | domestic/captive | captive | 4 | F2 Hybrid | yes |  |
| FSS020 | captive | zoo 12 | f | hair | WC2 | Fsi26 | domestic/captive* | captive | 6 | F2 Hybrid | yes |  |
| FSS021 | captive | zoo 17 | m | hair | DC1 | Fca1047A | domestic/captive | captive | 5 | F2 Hybrid | yes |  |
| FSS022 | captive | zoo 17 | f | hair | WC3 | Fsi257 | domestic/captive | captive | 3 | F2 Hybrid | yes |  |
| FSS023 | captive | zoo 19 | m | buccal swap | DC1 | Fca1047A | domestic/captive* | captive | 5 | F2 Hybrid | yes |  |
| FSS024 | captive | zoo 19 | f | buccal swap | DC1 | Fca1047A | domestic/captive | captive | 5 | F2 Hybrid | yes |  |
| FSS025 | captive | zoo 1 | m | hair | WC2 | Fsi26 | domestic/captive | captive | 4 | F2 Hybrid | yes |  |
| FSS026 | captive | zoo 1 | f | hair | WC2 | Fsi26 | domestic/captive | captive | 6 | F2 Hybrid | yes |  |
| FSS027 | captive | zoo 1 | m | hair | DC1 | Fca1047A | domestic/captive | captive | 5 | F2 Hybrid | yes |  |
| FSS028 | captive | zoo 7 | m | hair | DC1 | Fca1047A | domestic/captive | captive | 5 | F2 Hybrid | yes |  |
| FSS029 | captive | zoo 7 | f | hair | DC1 | Fca1047A | domestic/captive* | captive | 5 | F2 Hybrid | yes |  |
| FSS030 | captive | zoo 27 | f | hair | DC1 | Fca1047A | domestic/captive | captive | 5 | F2 Hybrid | yes |  |
| FSS031 | captive | zoo 28 | m | blood | DC1 | Fca1047A | domestic/captive | captive | 4 | F2 Hybrid | yes |  |
| FSS032 | captive | zoo 28 | f | blood | DC1 | Fca1047A | domestic/captive | captive | 3 | F2 Hybrid | yes |  |
| FSS034 | captive | zoo 27 | f | hair | DC1 | Fca1047A | domestic/captive | captive | 5 | F2 Hybrid | yes |  |
| FSS035 | captive | zoo 18 | m | tissue | WC2 | Fsi26 | domestic/captive | captive | 5 | F2 Hybrid | yes |  |
| FSS036 | captive | zoo 3 | f | buccal swap | DC1 | Fca1047A | domestic/captive | captive | 3 | Backcross Domestic | yes |  |
| FSS037 | captive | zoo 3 | m | buccal swap | DC1 | Fca1047A | domestic/captive | captive | 3 | F2 Hybrid | yes |  |
| FSS038 | captive | zoo 18 | ? | tissue | DC1 | Fca1047A | wild * | wild | 2 | F2 Hybrid | yes | 7) |
| FSS039 | captive | zoo 21 | f | hair | DC1 | Fca1047A | domestic/captive* | captive | 5 | F2 Hybrid | yes |  |
| FSS040 | captive | zoo 21 | m | hair | WC1 | Fsi246 | wild * | wild | 2 | F2 Hybrid | yes |  |
| FSS041 | captive | zoo 30 | m | hair | DC6 | - | domestic/captive | captive | 4 | F2 Hybrid | yes |  |
| FSS042 | captive | zoo 30 | f | hair | DC4 | - | domestic/captive* | wild | 4 | F2 Hybrid | yes |  |
| FSS043 | captive | zoo 4 | m | hair | DC1 | Fca1047A | domestic/captive | captive | 5 | F2 Hybrid | yes |  |
| FSS044 | captive | zoo 26 | m | hair | DC1 | Fca1047A | domestic/captive | captive | 5 | F2 Hybrid | yes |  |
| FSS045 | captive | zoo 26 | f | hair | DC1 | Fca1047A | domestic/captive | captive | 5 | F2 Hybrid | yes |  |
| FSS046 | captive | zoo 23 | ? | hair | DC1 | Fca1047A | domestic/captive | captive | 4 | Domestic | yes |  |
| FSS047 | captive | zoo 23 | ? | hair | DC1 | Fca1047A | domestic/captive* | captive | 5 | F2 Hybrid | yes |  |
| FSS048 | captive | zoo 20 | m | blood and hair | DC1 | Fca1047A | wild * | wild | 2 | F2 Hybrid | yes |  |
| FSS049 | captive | zoo 4 | f | hair | DC1 | Fca1047A | domestic/captive | captive | 5 | F2 Hybrid | yes |  |
| FSS050 | captive | zoo 3 | f | tissue | DC1 | Fca1047A | domestic/captive | captive | 3 | Backcross Domestic | yes |  |
| FSS051 | captive | zoo 3 | m | tissue | DC1 | Fca1047A | domestic/captive | captive | 3 | Backcross Domestic | yes |  |
| FSS052 | captive | zoo 22 | m | hair | DC1 | Fca1047A | domestic/captive | captive | 5 | Backcross Domestic | yes |  |
| FSS053 | captive | zoo 22 | f | hair | DC1 | Fca1047A | domestic/captive | captive | 5 | F2 Hybrid | yes |  |
| FSS054 | captive | zoo 10 | m | blood | WC2 | Fsi26 | domestic/captive | captive | 6 | F2 Hybrid | yes |  |
| FSS055 | captive | zoo 10 | f | blood | WC2 | Fsi26 | domestic/captive | captive | 6 | F2 Hybrid | yes |  |
| FSS056 | captive | zoo 10 | f | blood | WC2 | Fsi26 | domestic/captive | captive | 6 | F2 Hybrid | yes |  |
| FSS057 | captive | zoo 10 | f | blood | WC2 | Fsi26 | domestic/captive | captive | 6 | F2 Hybrid | yes |  |
| FSS058 | captive | zoo 10 | m | buccal swap | WC2 | Fsi26 | domestic/captive | captive | 6 | Backcross Domestic | yes |  |
| FSS059 | captive | zoo 10 | m | buccal swap | WC2 | Fsi26 | domestic/captive | captive | 6 | F2 Hybrid | yes |  |
| FSS060 | captive | zoo 3 | f | blood, buccal swap and hair | WC1 | Fsi246 | domestic/captive* | domestic | 4 | F2 Hybrid | yes |  |
| FSS061 | captive | zoo 3 | m | blood, buccal swap and hair | WC3 | Fsi257 | domestic/captive | captive | 3 | Backcross Domestic | yes |  |
| FSS062 | captive | zoo 2 | f | hair | DC1 | Fca1047A | domestic/captive | captive | 4 | F2 Hybrid | yes |  |
| FSS063 | captive | zoo 2 | m | hair | DC1 | Fca1047A | domestic/captive* | captive | 4 | F2 Hybrid | yes |  |
| FSS064 | captive | zoo 2 | f | hair | DC1 | Fca1047A | domestic/captive | captive | 4 | F2 Hybrid | yes |  |
| FSS065 | captive | zoo 2 | f | hair | DC1 | Fca1047A | domestic/captive | captive | 4 | F2 Hybrid | yes |  |
| FSS066 | captive | zoo 2 | f | hair | DC1 | Fca1047A | domestic/captive | captive | 4 | F2 Hybrid | yes |  |
| FSS067 | captive | zoo 13 | ? | hair | na | na | wild | wild | 2 | Wildcat | yes | 6) |
| FSS069 | captive | zoo 11 | f | hair | DC1 | Fca1047A | domestic/captive | captive | 5 | Domestic | yes |  |
| FSS070 | captive | zoo 5 | f | hair | WC2 | Fsi26 | domestic/captive | captive | 6 | F2 Hybrid | yes |  |
| FSS071 | captive | zoo 29 | f | blood and hair | WC1 | Fsi246 | domestic/captive* | captive | 5 | F2 Hybrid | yes |  |
| FSS072 | captive | zoo 5 | m | hair | WC2 | Fsi26 | domestic/captive* | captive | 6 | F2 Hybrid | yes |  |
| FSS074 | captive | zoo 16 | m | tissue | DC1 | Fca1047A | domestic/captive | captive | 5 | F2 Hybrid | yes |  |
| FSS075 | captive | zoo 16 | f | tissue | DC1 | Fca1047A | domestic/captive | captive | 5 | F2 Hybrid | yes |  |
| FSS076 | captive | zoo 6 | f | hair | DC1 | Fca1047A | domestic/captive | captive | 5 | F2 Hybrid | yes |  |
| FSS078 | captive | zoo 6 | m | hair | WC1 | Fsi246 | domestic/captive | captive | 6 | F2 Hybrid | yes |  |
| FSS077 | captive | zoo 6 | f | hair | WC2 | Fsi26 | domestic/captive | captive | 6 | F2 Hybrid | yes | 8) |
| FSS079 | captive | zoo 9 | m | hair | DC1 | Fca1047A | domestic/captive | captive | 4 | F2 Hybrid | yes |  |
| FSS080 | captive | zoo 24 | m | hair | DC1 | Fca1047A | domestic/captive | captive | 4 | Backcross Domestic | yes |  |
| FSS081 | captive | zoo 24 | m | hair | DC1 | Fca1047A | domestic/captive | captive | 4 | F2 Hybrid | yes |  |
| FSS082 | captive | zoo 24 | f | hair | DC1 | Fca1047A | domestic/captive | captive | 4 | F2 Hybrid | yes |  |
| DC001 | domestic | private J. Deppermann | m | hair | DC7 | Fca1914c | domestic/captive | domestic | 1 | F2 Hybrid | yes |  |
| DC002 | domestic | private K. Ociepka | m | hair | DC8 | - | domestic/captive | domestic | 1 | Domestic | yes |  |
| DC003 | domestic | private J. Hillen | f | hair | DC8 | - | domestic/captive | domestic | 1 | Domestic | yes |  |
| DC004 | domestic | private H. Strunk | m | hair | DC7 | Fca1914c | domestic/captive | domestic | 1 | Domestic | yes |  |
| DC005 | domestic | private H. Strunk | m | hair | DC2 | Fca4473 | domestic/captive | domestic | 1 | Backcross Domestic | yes |  |
| DC006 | domestic | private H. Strunk | m | hair | DC3 | Fca2700 | domestic/captive | domestic | 1 | Backcross Domestic | yes |  |
| DC007 | domestic | private H. Strunk | f | hair | DC1 | Fca1047A | domestic/captive | domestic | 1 | Backcross Domestic | yes |  |
| DC008 | domestic | private H. Strunk | f | hair | DC3 | Fca2700 | domestic/captive | domestic | 1 | F2 Hybrid | yes |  |
| DC009 | domestic | private H. Strunk | f | hair | DC3 | Fca2700 | domestic/captive | domestic | 1 | Domestic | yes |  |
| DC010 | domestic | private H. Strunk | f | hair | DC7 | Fca1914c | domestic/captive | domestic | 1 | Domestic | yes |  |
| DC011 | domestic | private K. Scharafin | f | hair | DC3 | Fca2700 | domestic/captive | domestic | 1 | Backcross Domestic | yes |  |
| DC012 | domestic | private K. Scharafin | f | hair | DC3 | Fca2700 | domestic/captive | domestic | 1 | Domestic | yes |  |
| DC013 | domestic | private K. Scharafin | f | hair | DC7 | Fca1914c | domestic/captive | domestic | 1 | Domestic | yes |  |
| DC014 | domestic | private K. Scharafin | m | hair | DC9 | Fca4240 | domestic/captive | domestic | 1 | Domestic | yes |  |
| DC015 | domestic | private K. Scharafin | m | hair | DC7 | Fca1914c | domestic/captive | domestic | 1 | Domestic | yes |  |
| DC017 | domestic | private S. Kettermann | m | hair | DC7 | Fca1914c | domestic/captive | domestic | 1 | Domestic | yes |  |
| DC018 | domestic | private J. Hillen | ? | hair | DC7 | Fca1914c | domestic/captive | domestic | 1 | Domestic | yes |  |
| DC019 | domestic | animal shelter Osterode | ? | hair | DC10 | - | domestic/captive | domestic | 1 | Domestic | yes |  |
| DC020 | domestic | animal shelter Osterode | ? | hair | DC3 | Fca2700 | domestic/captive | domestic | 1 | Domestic | yes |  |
| DC021 | domestic | animal shelter Osterode | ? | hair | DC7 | Fca1914c | domestic/captive | domestic | 1 | Backcross Domestic | yes |  |
| DC022 | domestic | animal shelter Osterode | ? | hair | DC1 | Fca1047A | domestic/captive | domestic | 1 | Domestic | yes |  |
| DC023 | domestic | animal shelter Osterode | ? | hair | DC7 | Fca1914c | domestic/captive | domestic | 1 | Backcross Domestic | yes |  |
| DC024 | domestic | animal shelter Osterode | ? | hair | DC3 | Fca2700 | domestic/captive | domestic | 1 | Backcross Domestic | yes |  |
| DC025 | domestic | animal shelter Osterode | ? | hair | DC7 | Fca1914c | domestic/captive | domestic | 1 | F2 Hybrid | yes |  |
| DC026 | domestic | animal shelter Osterode | ? | hair | DC7 | Fca1914c | domestic/captive | domestic | 1 | Backcross Domestic | yes |  |
| DC027 | domestic | animal shelter Osterode | ? | hair | DC7 | Fca1914c | domestic/captive | domestic | 1 | Domestic | yes |  |
| DC028 | domestic | animal shelter Osterode | ? | hair | DC7 | Fca1914c | domestic/captive | domestic | 1 | Domestic | yes |  |
| DC029 | domestic | animal shelter Osterode | ? | hair | DC7 | Fca1914c | domestic/captive | domestic | 1 | Domestic | yes |  |
| DC030 | domestic | animal shelter Osterode | ? | hair | DC7 | Fca1914c | domestic/captive | domestic | 1 | Backcross Domestic | yes |  |
| DC031 | domestic | animal shelter Osterode | ? | hair | DC7 | Fca1914c | domestic/captive | domestic | 1 | Domestic | yes |  |
| DC032 | domestic | animal shelter Osterode | ? | hair | DC7 | Fca1914c | domestic/captive | domestic | 1 | Backcross Domestic | yes |  |
| DC033 | domestic | animal shelter Osterode | ? | hair | DC3 | Fca2700 | domestic/captive | domestic | 1 | Domestic | yes |  |
